# Assessment of Referrals and Hospitalizations in the Hospital Transformed into COVID-19 Facility in Poland during the “Spring Wave” of the Epidemic in 2020—A Cross-Sectional Study

**DOI:** 10.3390/ijerph18137143

**Published:** 2021-07-03

**Authors:** Agnieszka Kasiukiewicz, Zyta Beata Wojszel

**Affiliations:** 1 Department of Geriatrics, Hospital of the Ministry of Interior in Bialystok, 15-471 Bialystok, Poland; wojszel@umb.edu.pl; 2 Department of Geriatrics, Medical University of Bialystok, 15-471 Bialystok, Poland

**Keywords:** COVID-19 treatment, healthcare system, COVID-19 hospital, COVID-19 patients characteristics

## Abstract

The study aimed to evaluate hospitalizations in a COVID-dedicated facility during the “spring wave” of the epidemic in 2020 in Poland and analyze changes in access to hospital treatment in the country in the early phase of the pandemic. We investigated all referrals and admissions to the Ministry of Interior and Administration hospital in Białystok from 14 April to 14 August 2020. A total of 238 patients were referred to the hospital (with a median age of 64.5 years; IQR, 44–78), most commonly with fever (*n* = 151; 63.5%). Only 135 (56.7%) were admitted (5.5% of the number hospitalized in the same period in 2019). SARS-CoV-2 was confirmed in 42 (17.7%) cases. Older people with concomitant diseases and disabilities dominated. Seventeen patients (12.6%) required ICU treatment, and 19 (14%) died. Based on the National Health Fund data, we also examined changes in the rate of hospitalizations in Poland and in selected Polish COVID/ and non-COVID hospitals between February and August 2019 and 2020. The number of hospitalizations in Poland decreased dramatically in comparison to 2019. A very low hospitalization rate, significantly lower than in structurally similar non-COVID hospitals, was observed in transformed hospitals. Better use of hospital resources was observed when the hospital was semi-transformed and had the flexibility to adapt to epidemiological needs. The study seems to confirm that the system of transformed COVID hospitals resulted in unused healthcare resources and limited patient access to medical services in the early period of the epidemic. As a consequence, systemic modifications allowing the maximization and adequate use of the Polish healthcare system’s limited resources have been implemented.

## 1. Introduction

The new strain of coronavirus—a severe acute respiratory syndrome coronavirus 2 (SARS-CoV-2)—emerged in Hubei Province, China, in 2019 [1]. It spread worldwide in the first months of 2020, resulting in the COVID-19 pandemic, declared by the World Health Organization (WHO) on 11 March 2020 [2].

The spread of the SARS-CoV-2 virus hit Poland in early March 2020. The first case of the disease was confirmed on 4 March 2020, and on 12 March, a lockdown was introduced across the country. Teaching in schools was suspended and mass events were canceled. Poland’s borders were closed for air and rail traffic on 15 March [3]. The Minister of Health declared an epidemic in Poland with a regulation on 20 March 2020 [4].

It was decided to base the organization of healthcare in Poland during the COVID-19 epidemic on a system of so-called “single-purpose infectious diseases hospitals” dedicated to patient care with this disease [5]. Twenty-two hospitals across the country were transformed this way. Patients suspected of COVID-19, with the confirmed disease, and only infected but requiring medical care for other reasons were referred to these facilities and infectious wards existing in some hospitals. At that moment, it was recommended to suspect COVID-19 in a patient who had at least one of the following four main symptoms: Fever, cough, shortness of breath, or a severe general condition not explained by any other cause [6]. This sudden change in the hospital care system organization was combined in Poland with a change in primary care organization, with a shift towards telemedicine solutions. These included, among other things, the increasingly widespread use of medical e-visits, electronic prescriptions, and e-referrals.

As the observed rates of COVID-19 were comparatively low in March and April, the gradual defrosting of the Polish economy began on 20 April. What is more, as at the beginning of June single-purpose hospital occupancy amounted to an average of approximately 40 percent [7], the Ministry of Health, together with the National Health Fund and voivodes, decided that these clinics would gradually return to their original, multi-profile functions.

In some experts’ opinions, the system based on COVID-19 dedicated hospitals was not an optimal solution. They pointed out that many hospital beds were frozen, which resulted in limited access to specialist hospital care for patients without a SARS-CoV-2 infection [8,9,10]. It has also been critically reviewed in terms of care provided to patients suspected of having an infection and with COVID-19.

The study aimed to evaluate the referrals and hospitalizations in a hospital transformed into COVID-19 facility during the “spring wave” of the epidemic in 2020 in Poland. Additionally, we intended to analyze changes in access to hospital treatment in the country during the study period and to evaluate the created model of the organization of care for patients infected and not infected with SARS-CoV-2 in Poland in this first period of epidemic through this prism.

## 2. Materials and Methods

### 2.1. The Cross-Sectional Study

#### 2.1.1. Patient and Setting Characteristics

The cross-sectional study included all patients referred to the Hospital of the Ministry of Interior and Administration in Białystok transformed into a single-purpose hospital for infectious diseases dedicated to treating patients with suspected or confirmed COVID-19 (so called ‘COVID—hospital’) in the period from 14 April to 14 August 2020. We analyzed the circumstances and reasons for referral to this hospital, the occurrence of symptoms that could suggest COVID-19, and the final diagnosis at discharge from the hospital.

The Hospital of the Ministry of Interior and Administration (MSWiA hospital) was one of two (next to a hospital in Lomza) “single-purpose” hospitals located in north-eastern Poland, Podlaskie Province, one of the country’s 16 provinces. The first case with a confirmed SARS-CoV-2 infection was found in this region on 17 March. Due to the low bed occupancy observed in the first months of the hospital’s new role, a decision was made on 1 June 2020, to return some of its departments to providing medical services to uninfected patients, leaving only several hospital beds for patients with COVID-19. Before the transformation, the hospital had 159 hospital beds and eight departments (Cardiology with the Laboratory of Hemodynamics and Electrotherapy, Gastroenterology, Geriatrics, General Surgery, Oncological Surgery, Orthopedics, Urology, and Intensive Care Unit with 9 beds) as well as several specialist outpatient clinics. After re-profiling and considering the safety requirements, the following wards were created: Observation Ward with nine isolation rooms, Infectious Ward with twenty-five beds, ICU with nine beds, and two Surgery Wards—for patients suspected of COVID-19 and with a confirmed COVID-19 infection.

#### 2.1.2. Measurements

The data were collected retrospectively based on patients’ electronic hospital health records. It included sociodemographic data (sex, age, place of residence), referral data (referring person, the leading diagnosis, the patient being in quarantine or already diagnosed with COVID-19, the source of infection), the presence of symptoms pointing to COVID-19 (fever on admission, cough, dyspnea, decreased saturation on admission, severe general condition), the presence of comorbidities that worsen the prognosis in COVID-19, further management of the patient (discharge home, transfer to another hospital, hospital admission), final diagnosis, the need for ICU treatment, and the final outcome (death, discharge home, referral to another facility). All COVID-19 positive cases were confirmed by real-time reverse transcriptase-polymerase chain reaction (RT PCR) assay from nasal and pharyngeal swabs or lower aspiratory tract aspirates [11,12].

### 2.2. Hospitalisation Rates Analysis

Based on the National Health Fund data [13], we also examined changes in the rate of hospitalizations in Poland and in selected COVID and non-COVID hospitals between February and August 2019 and 2020. For a comparative analysis of the number of hospitalizations in the consecutive months of 2019 and 2020, we chose two province hospitals, the Provincial Hospital in Bialystok (non-COVID facility) and the Hospital in Lomza (COVID-facility), and two university hospitals, the University Hospital in Cracow (semi-COVID facility) and the University Hospital in Bialystok (non-COVID facility). When choosing hospitals, we were guided by the similarity in terms of the size and structure of these centers. The university hospital in Cracow, operated in a different basis than other COVID-hospitals—it was a ‘semi-transformed’ hospital, which obtained the consent of the National Health Fund for flexible adaptation of its resources to the needs of providing services to patients with COVID-19 [14].

### 2.3. Statistical Analysis

The STATISTICA 13.3 software package (TIBCO Software, Palo Alto, CA, USA) was used for statistical analyses. The distribution of variables was checked with Shapiro–Wilk tests. They were presented as frequency and percentage, categorical variables, and as medians (Me) and interquartile range (IQR), continuous variables. Proportions were compared using χ^2^ tests, while the Mann–Whitney U test was used to compare medians. The U test for two ratios was used to compare percentage changes in number of hospitalizations between hospitals. In all analyses, a two-tailed *p* value of less than 0.05 was regarded as significant.

### 2.4. Ethics Approval

We obtained the hospital director’s consent to use medical data following the requirements of the General Data Protection Regulation (GDPR). All procedures performed in the study were following the Helsinki declaration and its later amendments. The study can be classified as a study of ‘usual practice’.

## 3. Results

### 3.1. COVID-Hospital Functioning Analysis—A Cross-Sectional Study

#### 3.1.1. Study Cohort Characteristics

In the study period, 238 patients, 94 (39.5%) women and 144 (60.5%) men, were referred to the studied hospital (Table 1). The median age was 64.5 years (IQR 44; 78), and 58.4% of patients were over 60 years of age. Most of the patients—172 people (72.3%)—were referred because of suspected SARS-CoV-2 infection. Thirty-four (14.3%) patients with an established COVID-19 diagnosis were referred from other hospitals or home isolation due to the worsening of their general condition. The other 32 patients (13.4%) admitted to the hospital required medical attention in quarantine.

Patients were most often referred by the emergency medical service (39.9%), transferred from other hospitals (10.5%), and infectious wards for specialist treatment such as surgery or invasive cardiology treatment (9.2%); less often, they were referred by general practitioners and specialist outpatient clinics (11.3% in total). Up to 18.9% of patients came to the emergency room (ER) on their own due to malaise; more often, they were above 60 years of age (30.3% vs. 10.8% in the older group). The primary referral diagnoses were fever (91 patients, 38.2%), COVID-19 suspected (ICD-10-U07), dyspnea, and pneumonia. Patients in quarantine required medical attention mainly due to injuries (17.7%), gastrointestinal symptoms (13.7%), stenocardia (11.7%), and other cardiological causes (7.8%).

#### 3.1.2. Main Symptoms That Could Suggest COVID-19

Fever was the most common symptom possibly indicating COVID-19. It was present in 151 (63.5%) people referred to the hospital (Me, 37.5 C degree; IQR, 36.7, 38.2), and in 82 patients, it was the only complaint. A cough was present in 53 (22.3%) and dyspnea in 75 (31.5%) patients in the latter group, and the median blood saturation (SatO2) was 93% (IQR, 88–97). Twenty-six (10.9%) patients were referred in a severe general condition. Fifty-four patients admitted to the hospital had no symptoms indicative of COVID-19—31 were referred during quarantine for other medical reasons and 23 patients were not in quarantine or isolation. However, they were referred to the COVID-19 hospital as suspected COVID patients (U07.2).

#### 3.1.3. Patient Transfer from the ER

**Figure 1:**
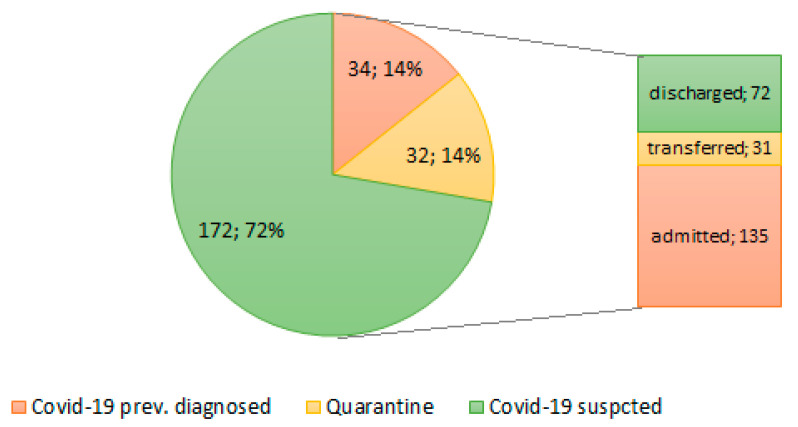
Characteristics and clinical decisions of patients referred to the COVID-hospital Emergency Room. Data are *n*; %.

After initial diagnosis and treatment, 72 patients were discharged from the ER/ isolation room. A total of 26 patients were transferred to other hospitals, and five patients were transferred to other COVID-hospitals with a different specialist profile (neurology, ophthalmology) (Figure 1).

#### 3.1.4. Characteristics of Hospitalized Patients

A total of 135 patients were admitted to the hospital (56.7% of referrals), including 25 with a previously diagnosed COVID-19 infection. They were mostly over 60 years old (76.3%). Ninety-nine patients were admitted to the observation ward, 20 to the infectious diseases ward, 8 to the intensive care ward, and 8 to the surgery ward.

SARS-CoV-2 infection was confirmed only in 42 (17.7%) patients reporting to the emergency room, 34 of whom had been diagnosed before the admission. Thirty-three (78.6%) of them were admitted to the hospital, two (4.8%) were transferred to other specialist centers, and seven (16.7%) were discharged for home isolation after the initial provision in ER. The final leading diagnoses in this group of patients were: COVID-19 pneumonia (83.3%), cardiovascular diseases (7.14%) and respiratory failure, injuries, limb ulcers, and renal failure requiring dialysis (2.4% each)

Older individuals burdened with cardiovascular diseases, diabetes, COPD, cancer, and undergoing immunosuppressive treatment dominated among the hospitalized (103, 74.1%). A total of 39% of them were disabled in daily activities and homebound, and 15% had dementia. Delirium episodes were observed in 23% of patients during hospitalization (Table 2).

**Figure 2:**
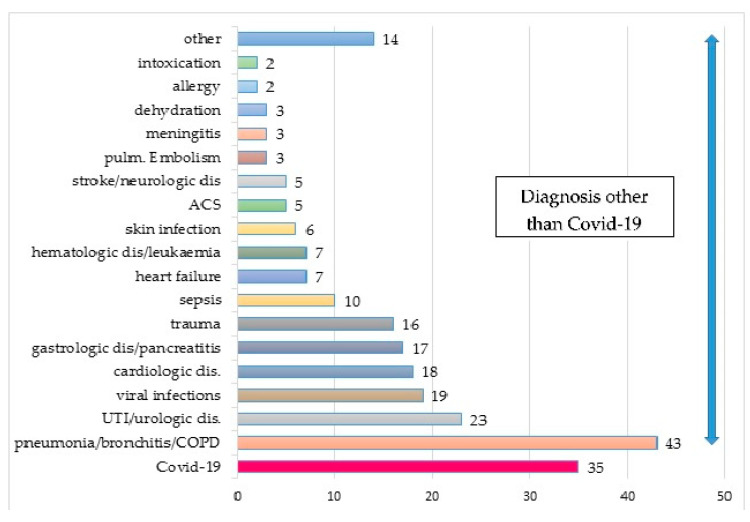
Final diagnoses of patients referred to the COVID-hospital (*n* = 238). Abbreviations: ACS, acute coronary syndrome; COPD, chronic obstructive pulmonary disease; UTI, urinary tract infection.

The final diagnosed reason for health deterioration as the leading cause of hospitalization was pneumonia or respiratory tract infection, including exacerbation of COPD (18.1%), urinary tract infection and urological causes (9.6%), cardiological diseases (12.6%), and gastrointestinal disorders (7.2%) (Figure 2).

In patients admitted with fever, the most common diagnosis was pneumonia and respiratory tract infection (21.6%), other viral infections (12.6%), and urinary tract infection (12.6%). A cough was mainly associated with pneumonia (50.9%), pulmonary embolism, and cardiological causes. Patients admitted with dyspnea were diagnosed with respiratory tract infection (30.7%) and cardiological causes (17.4%), including acute coronary syndromes and cardiovascular failure. Patients in severe general health conditions, apart from COVID-19, were diagnosed with sepsis, pneumonia, or myocardial infarction. Seventeen patients required treatment in the ICU-13 with diagnosed COVID-19 (39% of patients admitted with COVID-19) and four without a diagnosis of COVID (4% of patients COVID-19 negative)- Table 3.

A total of 19 patients (14% of hospitalized) died. Mortality in the group of hospitalized patients with diagnosed COVID-19 was 33% (11 people) and was statistically higher compared to patients hospitalized without COVID-19 (8%; *p* < 0.001). ICU mortality was the highest 58.8% (10 patients), and outside the ICU, it was equal to 7.6% (9 patients).

### 3.2. Changes in the Number of Hospitalizations in Poland and in Selected COVID- and Non-COVID Hospitals between 2019 and 2020—NHF Data Analysis

#### 3.2.1. Comparison of Hospitalization Numbers in Poland in 2019 and 2020

**Figure 3:**
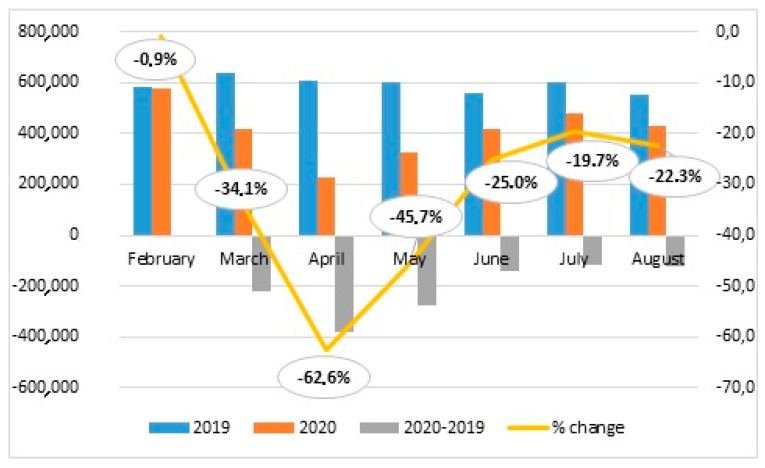
Number of hospitalizations in Poland in February–August period in the years 2019 and 2020 and % change between 2019 and 2020 in consecutive months.

Numbers of hospitalizations in Poland in the consecutive months of the study period (February–August 2019 and 2020) based on the National Health Fund data and the rate of change between 2019 and 2020 are presented on Figure 3. The number of hospitalizations in Poland in the analyzed period of 2020 decreased dramatically in comparison to 2019. The largest decrease in the number of hospitalizations compared to the previous year took place in April. Subsequently, the number of hospitalizations began to increase, but in the following months, their decline by about one-fifth continued.

#### 3.2.2. Hospitalization Rates in COVID- and Non-COVID Hospitals

A comparative analysis of the number of hospitalizations in the consecutive months of 2019 and 2020 in chosen COVID- and non-COVID hospitals is presented in Table 4.

In all the analyzed hospitals, large drops in the number of hospitalizations were observed in the subsequent months of 2020 compared to the same period of the previous year. The largest percentage decreases took place in April and May. A very low hospitalization rate, significantly lower than in structurally similar non-COVID hospitals, was observed in transformed hospitals. In the next months, the number of hospitalizations began to increase, but did not reach the 2019 level. However, in the case of the compared university hospitals, the percentage of decreases did not differ as much (although the differences were statistically significant) as in the case of decreases observed in county hospitals.

## 4. Discussion

This study illustrates the situation of hospital care in Poland during the first wave of the SARS-CoV-2 epidemic from the perspective of hospitalizations in one of 22 homonymous COVID-19-patients-dedicated hospitals. Single-purpose infectious diseases hospitals aimed to support sparse already existing infectious diseases wards in diagnosing and treating COVID-19 patients who suffer additional disorders. Simultaneously, they aimed to minimize the risk of virus transmission and prevent COVID-19-negative patients’ access to healthcare [15]. These hospitals were transformed following the example of similar solutions in other countries such as Fever Clinics in China, developed during the SARS-Cov1 epidemic and then improved [16,17,18]. In other countries, slightly different solutions have been adopted, e.g., the creation of temporary hospitals, as was done in Madrid [19]. However, the scale of pressure on healthcare in patients infected with Sars -Cov2 was much greater. Moreover, such solutions were dedicated only to infected patients, which was certainly associated with fewer organizational problems.

The network of single-purpose infectious diseases hospitals dedicated to COVID-19 patients was introduced in Poland during the sudden threat of the SARS-CoV-2 pandemic. The system was introduced in March of this year, i.e., when we were fearful of repeating the Italian or Spanish scenario [20,21] in our country. That is why no one then knew exactly how these centers were supposed to function in practice. Ultimately, however, the course of the epidemic in spring 2020 in Poland was not associated with many cases of infected individuals [22]. The number of hospitalized patients diagnosed with COVID-19 was much smaller compared to other countries, where temporary hospitals had to be created from non-medical facilities [19]. The transformed hospitals became reservoirs for patients not finally diagnosed with COVID-19, as confirmed by our analysis. Patients were referred to the ER with any symptom suggesting COVID-19, and even without “the leading” symptoms of COVID. This meant that patients were sometimes not admitted on time when they were supposed to. Acute coronary syndromes were treated for dyspnea; stroke was treated like a ‘severe health status condition’ at 37 °C. Our research results, analyzing the functioning of one of the hospitals transformed into COVID-19 dedicated facilities, seem to confirm this; five acute coronary syndrome cases were not confirmed as COVID-19, as well as two cases of stroke, intoxication, or epilepsy. The majority of referred patients were not confirmed as COVID-19, but rather as pneumonia, sepsis, urinary tract infections, or other viral infections. It is crucial to stress that the study was carried out at the beginning of the epidemic; hence, the first reactions to exposure to an unknown virus and the fear of infection and medical care should be considered [23].

On the other hand, this survey data showed that hospitalized patients were predominantly older and burdened with comorbidity, disability in ADL, and required interdisciplinary medical support—not only internal medicine, but also neurology, surgery, or intensive care. A similar characteristic of hospitalized COVID-19 patients was observed in other COVID-hospitals [15,24]. Some authors point out that concerning this population, an appropriate approach is needed to ensure the best quality of care and equal treatment, which on the one hand, would take into account functional limitations, but above all, biological and not calendar age [25].

As the analysis of the first months of the COVID-19 epidemic in Poland showed, most infected patients were located in big cities and the center and south of the country [21]. The surveyed COVID-hospital was located in a region with a relatively low percentage of COVID-19 infections—until 14 August, there were 55,312 cases in Poland, and in Podlasie, only 1133 diagnosed cases [26]. Therefore, the hospital’s therapeutic potential was not fully used during this time. In the analyzed period of 2020, the total number of patients admitted to the hospital decreased significantly—only 135 patients were hospitalized during four months, while in the corresponding period of 2019, there were 2444. It was only 5.5% of the regular hospital occupancy and thus non-use of the hospital’s therapeutic potential. The transformation resulted in limited access to specialized medical services. Scheduled patient admissions for invasive cardiological procedures, urological, orthopedic, and oncological surgery had to be abandoned. It resulted in an outflow of patients and specialist staff. In the initial period, the specialist clinics were also closed, and outpatient care was limited. The observation hospital unit was full almost all the time, but most of the patients turned out to be COVID-negative. Out of the admitted patients, 57 were transferred to other hospitals with a negative SARS-CoV-2 result, 19 died, and 59 were discharged home (in 7 cases—16% of those discharged home—they were treated in the observation department, as other hospitals refused to admit them, and thus beds were occupied).

In principle, the transformed hospitals were intended for COVID-plus patients. However, they partly became observation facilities, making access to hospital beds for the infected difficult if their number increased. It resulted from the lack of adaptation of other facilities to diagnosing patients with SARS-CoV-2 infection and the widespread panic and fear of an unknown virus. The centralization of care for infected individuals also caused significant logistical problems in the area of overloaded medical transport. Involved in the transport of infected people, it was not available in other acute medical conditions.

Adverse impact in the treatment of non-COVID-19 patients, a decreased number of diagnosed acute coronary syndromes, strokes, and delay in diagnosis of life-threatening disorders, including oncologic diagnoses, is an additional negative result of the SARS-CoV-2 pandemic [27,28]. This was reflected in the dramatic decline in the number of hospitalizations throughout Poland, not only in hospitals transformed into COVID-facilities, but also in those that should provide care to patients without such an infection. The reasons for this decline are complex. They could include not only organizational problems in healthcare, making access to medical facilities more difficult, but also patients’ fear of hospitalization, and contact with medical workers influenced that [29], especially as media reports confirmed the emergence of COVID-19 outbreaks in medical facilities. Although the number of hospitalizations began to increase gradually, their decline by about one-fifth continued.

A comparison of the situation in selected COVID- and non-COVID hospitals showed that in all the hospitals examined, large drops in the number of hospitalizations were observed in the analyzed months of 2020 compared to 2019. The drop in the number of hospitalizations was significantly more visible in structurally similar COVID-facilities than in non-COVID ones. This may confirm that the solution with transformed hospitals with low incidence of this disease turned out to be a redundant one. The increase in the use of hospital beds in the following months was by far greater in non-COVID hospitals, while facilities such as the hospital analyzed in this study were still blocked for potential COVID-19 patients. Better use of hospital resources was observed in a semi-transformed hospital (the University Hospital in Cracow) that had the flexibility to adapt to epidemiological needs. The analyzed hospital in Cracow can be treated as a semi-transformed facility rather than a full-transformed one, and it operated differently than other COVID-hospitals in Poland, flexibly adapting to the epidemiological situation and to the needs of providing services to patients with COVID-19. It was only in August, when the epidemiological situation in Cracow and Mazowieckie Voivodeship (pProvince) began to worsen, that the percentage difference increased visibly [26].

Although the Ministry of Health upheld the validity of the concept of one-purpose infectious hospitals, experts questioned it and, based on these experiences, were against keeping the system in its previous formula. They began to point out that it would be significantly more useful to create a “COVID” section in each hospital so that patients would not waste time looking for specialists in other facilities. They admitted that the idea of creating a COVID-dedicated hospital system was appropriate, but its implementation and the organization of this network were imperfect [9]. We have to admit that the study’s limitation lies in the fact that the more in-depth analysis covered the data of only one COVID-hospital. On the other hand, each region carries its own specificity. Compared to the rest of the country, the region in which the hospital in question is located was characterized by a relatively small number of COVID-19 cases, which undoubtedly impacted the results obtained. Research including all the transformed hospitals would unquestionably provide a complete picture of the situation. Nevertheless, the analysis of rates of hospitalizations in other COVID-hospitals seem to confirm these trends.

When analyzing the information on the number of hospitalizations in the entire country and in other selected hospitals, it should be taken into account that the statistical data provided by the National Health Fund has its limitations. Hospitalizations in individual hospitals are grouped according to the category of “homogeneous groups of patients”, according to which the fund pays for the services provided by the hospital. They do not take into account, for example, hospitalizations due to mental illnesses and in intensive care units—here, the settlement is based on the number of persons to days spent by patients in hospital. The small number of cases in the analyzed COVID-hospital may also be treated as a flaw of the study; however, it shows the negative aspects of the hospital’s transformation into a COVID-19 facility and “lockdown” of all existing departments when the number of reported COVID-19 cases is relatively low. During the second (autumn) wave of the epidemic, Poland’s healthcare was based on the three levels of the medical care model [30]. All hospitals with isolation rooms for suspected patients constitute the first level of care. The second level is represented by infectious diseases hospitals treating COVID-19 as the primary disorder. The third level of care—interdisciplinary specialist hospitals—is designated for treating other disorders in COVID-positive patients. The second wave shows that the number of infected patients is much higher, with significant treatment requirements in the intensive care unit, and burdened with high mortality [31]. Varying coordinated medical and epidemiological care strategies were needed, and the proposed new scheme for the functioning of the healthcare system seems to be an improved solution.

## 5. Conclusions

The study seems to confirm that the system of single-purpose infectious diseases hospitals for COVID-19 created during the early phase of the pandemic of the SARS-CoV-2 coronavirus epidemic was a redundant solution resulting in unused healthcare resources and limited patient access to medical services. The relatively small number of COVID-19 cases during this period certainly contributed to this outcome. Nevertheless, the observed unfavorable phenomena related to such a reorganization of the healthcare system should encourage healthcare decision-makers to make appropriate modifications allowing the maximization and adequate implementation of the Polish healthcare system’s insufficient resources. It seems that such organization of hospitals, enabling their flexible adaptation to epidemiological needs in the areas served by them, is the optimal solution.

## Abbreviation

ACS—acute coronary syndrome, ADL—activities of daily living, CAD—coronary artery disease, COPD—chronic obstructive pulmonary disease, ER—emergency room, GDPR—General Data Protection Regulation, IADL—instrumental activities of daily living, ICU—intensive care unit, IQR—interquartile range, Me—median, MSWiA—The Ministry of Interior and Administration, NHF—the National Health Fund, RT PCR—real-time reverse transcriptase-polymerase chain reaction, SARS-CoV-2—severe acute respiratory syndrome coronavirus 2, UTI—urinary tract infection, WHO—World Health Organization

## Figures and Tables

**Table 1:** Referrals to the COVID- hospital.

	Total	<60 Years	≥60 Years	P ^a^
Number of referrals	238 (100.0)	99 (41.6)	139 (58.4)	
Age, *years*, Me (IQR)	64.5 (44–78)	41 (33;50)	76 (67;84)	0.001 ^b^
Sex, *men*	144 (60.5)	67 (67.7)	77 (55.4)	0.06
Cause of the referral				
Fever	91 (38.2)	31 (31.3)	60 (43.2)	0.06
Dyspnoea	21 (8.8)	8 (8.1)	13 (9.4)	0.72
Respiratory Insufficiency	2 (0.8)	-	2 (1.4)	0.23
COVID-19	32 (13.5)	11 (11.1)	21 (15.1)	0.37
Pneumonia	21 (8.8)	6 (6.1)	15 (10.8)	0.21
Pulmonary disorders	6 (2.5)	2 (2)	4 (2.9)	0.66
Cardiological disorders	9 (3.8)	2 (2)	7 (5)	0.23
Acute coronary syndrome	5 (2.1)	2 (2)	3 (2.2)	0.92
Stenocardy	7 (2.9)	6 (6.1)	1 (0.7)	0.015
Trauma	14 (5.9)	12 (12.1)	2 (1.4)	0.005
Gastrointestinal disorders	17 (7.1)	13 (13.1)	4 (2.9)	0.003
Urological disorder	5 (2.1)	1 (1)	4 (2.9)	0.31
Neurological disorder	3 (1.3)	2 (2)	1 (0.7)	0.37
Hematological disorder	1 (0.4)	1 (1)	-	0.23
Intoxication	1 (0.4)	1 (1)	-	0.23
Dermatological disorder	2 (0.2)	-	2 (1.4)	0.19
Who referred				
Emergency medical Services	95 (39.9)	34 (34.3)	61 (43.9)	0.14
Infectious disease department	22 (9.2)	6 (6.1)	16 (11.5)	0.16
Hospital emergency ward	11 (4.6)	4 (4)	7 (5)	0.71
Other hospital	25 (10.5)	10 (10.1)	15 (10.8)	0.86
GP/ outpatient department	27 (11.3)	7 (7.1)	20 (14.4)	0.08
Sanitary and epidemiological department	13 (5.5)	8 (8.1)	5 (3.6)	0.13
Patients presented themselves	45 (18.9)	30 (30.3)	15 (10.8)	0.002
Symptoms indicative of COVID-19				
Fever	151 (63.5)	56 (56.6)	95 (68.4)	0.06
Cough	53 (22.3)	18 (18.2)	35 (25.2)	0.2
Dyspnoea	75 (31.5)	18 (18.2)	57 (41)	<0.001
Severe general health status	26 (10.9)	6 (6.1)	20 (14.4)	0.04

Data are *n* (%), unless otherwise stated. ^a^ χ^2^ test or Fisher exact test, as appropriate; ^b^ U Mann–Whitney test. Abbreviations: GP, general practitioner; IQR, inter quartile range; Me, median.

**Table 2:** Characteristics of hospitalized patients.

Total	Total	<60 Years	≥60 Years	P ^a^
Admitted to the hospital	135 (59.3)	32 (32.3)	103 (74.1)	<0.001
Comorbidity:				
Hypertension	95 (70)	9 (28)	86 (83)	<0.001
CAD	33 (24)	-	33 (32)	<0.001
Atrial fibrillation	35 (26)	2 (6)	33 (32)	0.003
Heart failure	67 (50)	2 (6)	65 (63)	<0.001
Diabetes	46 (34)	5 (16)	41 (40)	0.01
COPD	20 (15)	2 (6)	18 (17)	0.12
Immunosuppressive therapy	14 (10)	2 (6)	12 (12)	0.38
Neoplasm	18 (13)	2 (6)	16 (16)	0.36
Dementia	15 (11)	-	15 (15)	0.02
IADL disability	43 (33)	4 (13)	39 (39)	0.01
Delirium during hospitalization	31 (23)	7 (22)	24 (23)	0.87
Transfer to Intensive care unit	17 (13)	3 (10)	14 (14)	0.55
Death	19 (14)	4 (13)	15 (15)	0.86

Data are *n* (%). ^a^ χ^2^ test or Fisher exact test, as appropriate; Abbreviations: CAD, coronary artery disease; COPD, chronic obstructive pulmonary disease; IADL, instrumental activities of daily living.

**Table 3:** Final diagnoses categorized by main COVID-19 symptoms.

Final Diagnoses	Coexisting Symptom by Admission			
	Fever (*n* = 151)	Cough (*n* = 53)	Dyspnoe (*n* = 75)	Severe Health Status (*n* = 26)
COVID-19 pneumonia	18 (11.9)	14 (26.4)	19 (25.3)	8 (30.8)
COVID-19 negative				
Pneumonia/bronchitis/COPD	33 (21.6)	27 (50.9)	23 (30.7)	3 (11.5)
Viral infections	19 (12.6)	-	1 (1.3)	-
Sepsis	10 (6.6)	1 (1.9)	4 (5.3)	4 (15.4)
UTI/urologic dis	19 (12.6)	-	2 (2.7)	-
Heart failure	2 (1.3)	1 (1.9)	5 (6.7)	-
ACS	1 (0.7)	-	5 (6.7)	2 (7.7)
Pulmonary embolism	3 (2)	2 (3.8)	1 (1.3)	1 (3.9)
Cardiologic dis	5 (3.3)	1 (1.9)	2 (2.7)	1 (3.9)
Trauma	3 (2)	-	-	-
Gastrologic dis	8 (5.3)	1 (1.9)	1 (1.3)	-
Hematologic dis/leukaemia	4 (2.7)	2 (3.8)	2 (2.7)	-
Dehydration	2 (1.3)	-	2 (2.7)	1 (3.9)
Skin infections	5 (3.3)	1 (1.9)	1 (1.3)	-
Meningitis	3 (2)	-	1 (1.3)	1 (3.9)
Neurologic dis/stroke	4 (2.7)	1 (1.9)	-	1 (3.9)
Intoxication	2 (1.3)	-	-	-
Allergy	1 (0.7)	-	1 (1.3)	-
other	9 (5.9)	2 (3.8)	5 (6.7)	4 (15.4)

Data are *n* (%). Abbreviations: COPD, chronic obstructive pulmonary disease; ACS, acute coronary syndrome; UTI, urinary tract infection.

**Table 4:** Comparison of number of hospitalizations in chosen Polish province and university “COVID” and “NON-COVID” hospitals (February–August period) in 2019 and 2020, and % change between 2019 and 2020 in consecutive months.

Hospitalizations				Months			
	February	March	April	May	June	July	August
COUNTY HOSPITALS							
COVID: Hospital in Lomza							
Number in 2019	1488	1537	1488	1512	1365	1474	1379
Number in 2020	1494	689	69	38	247	601	580
Change in number (2019–2020)	6	−848	−584	−1474	−1118	−873	−799
% change (2019–2020)	0.4	−55.2	−94.8	−97.5	−81.9	−59.2	−57.9
NON-COVID: Sniadecja Hospital in Bialystok							
Number in 2019	1933	1922	1890	1959	1776	2001	1601
Number in 2020	1810	1402	875	1395	1592	1747	1446
Change in number (2019–2020)	−123	−520	−1015	−564	−184	−254	−155
% change (2019–2020)	−6.4	−27.1	−53.7	−28.8	−10.4	−12.7	−9.7
P^a^	<0.001	<0.001	<0.001	<0.001	<0.001	<0.001	<0.001
UNIVERSITY HOSPITALS							
COVID: University Hospital in Cracow							
Number in 2019	4998	5168	4828	5189	4541	5449	4340
Number in 2020	4462	2982	1226	1887	2766	3636	1258
Change in number (2019–2020)	−536	−2186	−3602	−3302	−1775	−1813	−3082
% change (2019–2020)	−10.7	−42.3	−74.6	−63.6	−39.1	−33.3	−71.0
NON-COVID: University Hospital in Bialystok							
Number in 2019	3288	3713	3622	3468	3315	3520	2907
Number in 2020	3399	2226	961	1572	2150	2537	1990
Change in number (2019–2020)	111	−1487	−2661	−1896	−1165	−983	−917
% change (2019–2020)	3.4	−40.0	−73.5	−54.7	−35.1	−27.9	−31.5
P^b^	<0.001	0.03	0.25	<0.001	<0.001	<0.001	<0.001

P^a^—U-test for the comparison of % change (2019–2020) between COVID and NON-COVID county hospitals; P^b^—U test for the comparison of % change (2019–2020) between COVID and NON-COVID university hospitals. MSWiA, the Ministry of Interior and Administration.

## Data Availability

The data supporting the results in the current study are available from the corresponding author on reasonable request.
